# A simple procedure for directly obtaining haplotype sequences of diploid genomes

**DOI:** 10.1186/s12864-015-1818-4

**Published:** 2015-08-28

**Authors:** Harry A. Noyes, Derek Daly, Ian Goodhead, Suzanne Kay, Steven J. Kemp, John Kenny, Ilik Saccheri, Robert D. Schnabel, Jeremy F. Taylor, Neil Hall

**Affiliations:** Centre for Genome Research, Institute of Integrative Biology, University of Liverpool, Crown St., Liverpool, L69 7ZB UK; Division of Animal Sciences, University of Missouri, Columbia, Missouri USA

**Keywords:** Haplotype, Next generation sequencing, Cow, Scaffold, Protocol, Diploid genome sequence

## Abstract

**Background:**

Almost all genome sequencing projects neglect the fact that diploid organisms contain two genome copies and consequently what is published is a composite of the two. This means that the relationship between alternate alleles at two or more linked loci is lost. We have developed a simplified method of directly obtaining the haploid sequences of each genome copy from an individual organism.

**Results:**

The diploid sequences of three groups of cattle samples were obtained using a simple sample preparation procedure requiring only a microscope and a haemocytometer. Samples were: 1) lymphocytes from a single Angus steer; 2) sperm cells from an Angus bull; 3) lymphocytes from East African Zebu (EAZ) cattle collected and processed in a field laboratory in Eastern Kenya. Haploid sequence from a fosmid library prepared from lymphocytes of an EAZ cow was used for comparison. Cells were serially diluted to a concentration of one cell per microlitre by counting with a haemocytometer at each dilution. One microlitre samples, each potentially containing a single cell, were lysed and divided into six aliquots (except for the sperm samples which were not divided into aliquots). Each aliquot was amplified with phi29 polymerase and sequenced. Contigs were obtained by mapping to the bovine UMD3.1 reference genome assembly and scaffolds were assembled by joining adjacent contigs that were within a threshold distance of each other. Scaffolds that appeared to contain artefacts of CNV or repeats were filtered out leaving scaffolds with an N50 length of 27–133 kb and a 88–98 % genome coverage. SNP haplotypes were assembled with the Single Individual Haplotyper program to generate an N50 size of 97–201 kb but only ~27–68 % genome coverage. This method can be used in any laboratory with no special equipment at only slightly higher costs than conventional diploid genome sequencing. A substantial body of software for analysis and workflow management was written and is available as supplementary data.

**Conclusions:**

We have developed a set of laboratory protocols and software tools that will enable any laboratory to obtain haplotype sequences at only modestly greater cost than traditional mixed diploid sequences.

## Background

The low cost and high throughput of next generation sequencing has led to numerous reference genomes and resequenced genomes being published [1]. However, almost all of these projects neglect the fact that diploid organisms contain two genome copies and consequently what is published is a composite of the two. This means that the relationship between alternate alleles at two or more linked loci is lost. Humans typically carry 250–300 loss of function variants most of which exist as heterozygotes [2]. If two deleterious mutations are both heterozygous within the same gene and the deleterious alleles are both on the same chromosome then the individual will still have one non-affected copy of the gene and there may be no phenotypic consequence. However, if the individual is a compound heterozygote and both gene copies contain a deleterious allele then the activity of the gene product may be severely compromised, with detectable phenotypic consequences.

The phase relationship between common alleles can be recovered using population or pedigree data based on linkage disequilibrium or co-segregation, respectively. However, it is becoming increasingly apparent that a substantial amount of the phenotypic variation within populations is attributable to rare genomic variants [2] and the haplotypes on which these variants lie cannot be reliably inferred from genotypes at common marker single nucleotide polymorphisms (SNP). Diploid genome sequences have been published using a range of techniques; however, with the exceptions of [3] and [4] they require either large amounts of data, complex sample preparation or purpose built equipment, placing the method beyond the reach of most laboratories.

There have been multiple reports of sequencing from single cells [5–9] but few have gone on to sequence single chromosomes. The simplest strategy is to use single sperm. Individual sperm have been separated on a custom-built microfluidic device for whole genome amplification and sequencing [10]. The use of haploid gametes considerably simplifies the complexity of downstream data analysis and provides access to whole chromosome haplotypes, however gametes cannot practically be recovered from females and are difficult to obtain from many male animals. For example, males of many livestock species are castrated or slaughtered before sexual maturity and even intact bulls require training to donate semen for artificial insemination and consequently semen is usually only available from elite animals. Individual chromosomes from diploid cells have also been recovered and amplified using a custom built microfluidic device; however, the required equipment is currently restricted to a single laboratory [11].

Haploid sequences have been obtained by sequencing DNA diluted to less than a haploid genome per aliquot [1–3]. However Kaper et al. [3] acknowledged the difficulty of obtaining accurate dilutions at very low concentrations. Furthermore, the extraction, dilution and necessary mixing are likely to shear the DNA leading to short scaffolds, which requires more data for assembly and results in a greater difficulty in phasing across repeats or extended regions of homozygosity where there are no informative SNP for joining scaffolds. Regions of homozygosity are particularly likely in livestock where large numbers of animals are bred from semen from elite sires leading to significant levels of inbreeding. We have developed a single cell isolation protocol that allows the accurate selection of appropriately diluted DNA whilst minimizing the number of pipetting steps that might shear the DNA. This approach overcomes many of the difficulties in the method of Kaper et al. [3] and is potentially automatable using cell sorting devices. We have also developed a collection of open source Java and Perl software for processing the data and obtaining a rich set of metadata and quality metrics (Supplementary Data; Analysis Pipeline).

We tested the method with three sets of samples: 1) diploid sequence of an Angus steer using 75 bp fragment reads that was validated against Illumina BovineHD SNP (770 K) haplotypes imputed by Beagle 3.3.2 [12] without using pedigree information; 2) the diploid sequence of an Angus bull with DNA obtained from sperm using 75 bp fragment reads; 3) partial diploid sequences of 18 East African Zebu cattle obtained from 21 libraries of 2 × 100 bp paired end reads to discover common haplotypes. Finally, the diploid sequence of a single East African Zebu (EAZ) cow obtained from 2 × 100 bp paired-end reads from 47 fosmid pools was also obtained to validate the software [13, 14].

## Results and Discussion

### Isolation and amplification of aliquots of single cells

#### Diploid sequence of an angus steer

Analysis pipelines were developed using the 75 bp fragment reads for the Angus steer. The following results are from those data unless otherwise stated.

**Fig. 1:**
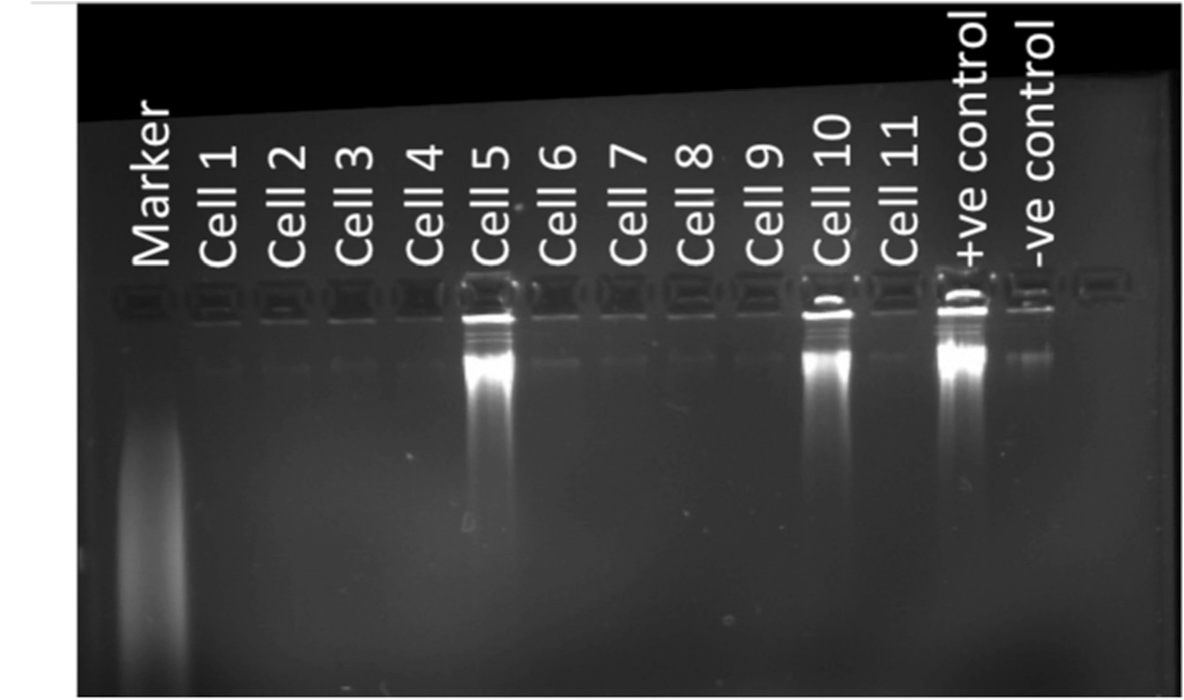
Genomiphi products from test amplifications from 1/6th of the lysate of an individual cell. Amplifications gave either clear negatives or clear positives

Forty aliquots each predicted to contain a single cell were prepared from the Angus steer 72711. Seven of the 40 test Genomiphi amplifications produced readily visible products on an agarose gel. No DNA was detectable by agarose gel electrophoresis in the remaining reactions (Fig. 1). The lower than expected proportion of positive reactions suggests that the cells were more diluted than predicted by counting on the haemocytometer and that therefore there was even less chance than expected of two cells being in the same reaction. The remaining lysate from the seven positive samples was divided into five aliquots and amplified with Genomiphi. Thirty of these 35 reactions had products that were detectable by fluorescence spectroscopy (Qubit, Life Technologies) with a mean concentration of 33 ng/μl (range 2–394 ng/μl).

A second Genomiphi reaction was performed on the Genomiphi product of all positive samples and this product was used to screen the genome by PCR at 24 loci to estimate the proportion of the genome that was represented in each sample. There was a mean of 6.0 PCR positive loci (σ = 4.3, range 0–21) for each subcellular sample. There was no correlation between the concentration of the Genomiphi product from a subcell and the number of PCR positive loci (r^2^ = 0.05). The observed mean of 6 positive loci is less than the expected mean of eight, which would represent 1/3rd of the haploid genome (1/6th of the diploid genome). This may be a consequence of the dropout of genomic regions that is frequently observed when using phi29 to amplify trace amounts of DNA but is consistent with the subcellular samples being derived from the DNA of less than a single cell. Thirty-one subcellular samples that produced between 3 and 14 positive PCR reactions were chosen for library preparation and sequencing to obtain 75 bp fragment reads on an ABI 5500. A bulk genomic DNA sample was also sequenced to obtain 2 × 100 bp paired end reads on an Illumina GAII instrument.

The semen sample from the Angus bull was diluted in the same way as the lymphocytes and 20 aliquots each expected to contain a single cell were amplified with Genomiphi. Seventeen of the 20 reactions generated detectable product and nine of these were selected for sequencing together with a sample of DNA amplified from 50 cells.

#### Sequence data

The expected Genomiphi product length from whole genomic DNA was > 10 kb and this was observed in agarose gels of Genomiphi products suggesting that degradation of chromosomal DNA during cell lysis and dilution had not reduced its mean size to less than 10 kb.

In order to create a reference with best possible mapping characteristics the 2 × 100 bp paired end reads of the bulk genomic DNA sample were first mapped to UMD3.1 bovine genome assembly with BWA and SNP were called using GATK. A modified reference sequence was then generated with IUPAC redundancy codes for heterozygous SNP using bcftools “consensus.” Reads from the subcellular libraries were mapped to the modified UMD3.1 assembly using BWA. Contigs of overlapping mapped reads were obtained with the purpose written Picard Module “ContigMetrics.” This showed that each subcellular library covered a mean of 14.2 % of the genome. This is less than the expected 33 % (2 × 1/6th) suggesting that the remaining 19 % of the genome was in regions derived from both chromosomes, not sequenced due to low depth of coverage for each library or lost to drop-outs in the amplification or during the sample preparation process.

**Fig. 2:**
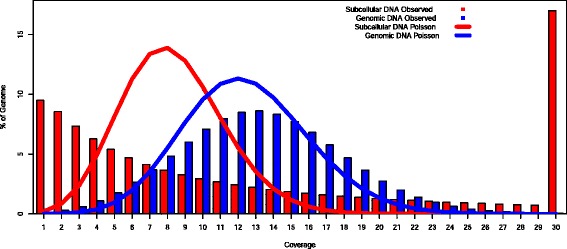
Histograms of read coverage of 75 bp fragment reads from the angus steer obtained by summing coverage data from all 31 subcellular libraries of diploid cells and separately for the bulk genomic library. The histogram shows the percentage of the genome at different coverages between one and 29 X. The observed coverage is shown as columns, whilst the expected random Poisson distribution of coverage with mean derived from the data is shown as a line. The observed coverage was truncated at 29 X for clarity, but 17 % of the genome had coverage > 29 X. The coverage of the bulk genomic DNA is only slightly broader than expected from the Poisson distribution, but coverage from fractions of cells is highly skewed

Phi29 amplification from trace amounts of DNA is expected to give rise to very uneven coverage with some regions having very high coverage and others very low or none [7]. The coverage distribution was obtained by summing the coverage distributions for all 31 subcellular libraries and was compared to the expected Poisson distribution. The two distributions differed substantially, with many more regions with low and high coverage observed in the data than expected from a Poisson distribution with the same mean (Fig. 2).

**Fig. 3:**
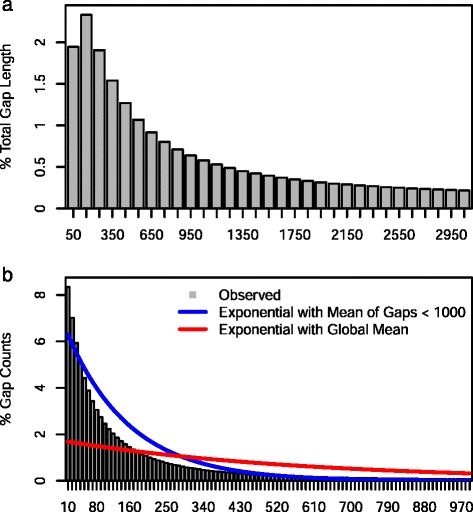
Histograms of lengths and counts of gaps between contigs. Note the 4-fold difference in X axis scale of the two plots: gap lengths are in bins of 100 bp and gap counts are in bins of 20 bp. Panel **a** shows the first 20 % of gap lengths (<3050 bp) and panel **b** the first 92 % of gap counts (<1000 bp). Panel **b** also shows the expected distribution of gap counts if they were exponentially distributed first using the global mean gap length of 584 bp and second using the mean length of gaps < 1000 bp (144 bp)

Contigs from diploid cells had an N50 length of 359 bp and a read coverage of 7.7 X. Given the low and skewed genome coverage (Fig. 2), it was expected that clusters of contigs would be observed with small gaps between contigs from a single amplicon and large gaps between contigs mapping to adjacent amplicons. This would give rise to many short gaps between contigs from the same amplicon and large gaps between contigs from adjacent amplicons. Groups of contigs could then be classified as scaffolds. Figure 3 shows histograms of percentages of total gap length in bins of 100 bp and of percentages of the total gap count in bins of 10 bp. Eighty percent of the total gap length is in gaps > 3000 bp but 50 % of gaps are less than 80 bp. This shows the expected skewed gap distribution with many small gaps between contigs from the same amplicon and large gaps between contigs from different amplicons. However, there was no clear discontinuity in the distributions that could be used to define a threshold gap length below which contigs could be joined into scaffolds. The problem was therefore to identify a rational strategy for joining contigs into scaffolds.

Gaps between contigs were assigned to 1000 bp bins and the mean gap length in each bin was obtained. For bins with gap lengths > 1000 bp the mean gap length was close to the midpoint of the bin size (expected 500 bp; observed 489 ± 6.8 bp), however the mean length of gaps that were < 1000 bp was 144 bp (σ = 175 bp). Therefore it appeared that gaps greater than 1000 bp were approximately randomly distributed and those that were less than 1000 bp were highly skewed in length.

If junctions of contigs and gaps were randomly distributed they would be expected to follow a Poisson Distribution [15]. Block lengths would then be expected to follow the inter arrival distances represented by a Poisson process which are exponentially distributed [16]. Figure 3b shows that the number of small gaps exceeded the numbers expected under an exponential distribution. The number of large gaps also exceeds the numbers expected under an exponential distribution. There were more gaps with lengths greater than 3424 bp than expected using the global mean gap length of 584 bp and more gaps with lengths greater than 468 bp than expected using the mean length of gaps < 1000 bp (data not shown). These deviations are consistent with the expectation that contigs will be clustered with short gaps between them and that clusters of contigs will have larger distances between them. This justifies the joining of clusters of contigs into scaffolds.

**Fig. 4:**
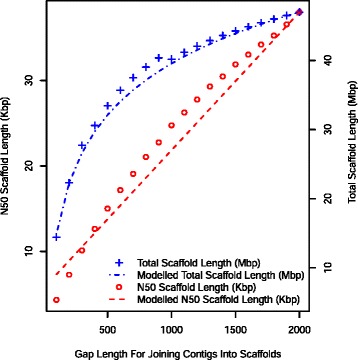
Effect of using different maximum gap lengths for joining contigs into scaffolds for chromosome 10. N50 scaffold lengths increase almost linearly (*y* = 16.9× + 5962; r^2^ = 0.98), whilst total length of scaffolds grows logarithmically (*y* = 1.09 × 10^7^ × ln(×/100) + 1.5 × 10^7^; r^2^ = 0.99) as the size of scaffolds being joined gets larger relative to the gaps between them. Note that N50 Scaffold Length (Kbp) is plotted against the left hand Y axis and Total Length of Scaffolds (Mbp) per library against the right hand Y axis

The sensitivity of N50 scaffold length and total length of all scaffolds to variation in the maximum gap length for joining adjacent contigs into scaffolds was tested using threshold gap lengths from 100 to 2000 bp in increments of 100 bp for chromosome 12 (Fig. 4). The N50 scaffold length increased almost linearly with increasing threshold gap length, however the rate of increase in total length of all scaffolds from a library declined as gap length increased, particularly after about 600 bp. This is presumably a consequence of a decline in the length of gap relative to the length of contig and scaffolds as more scaffolds are joined. The effect on N50 scaffold size of using an absolute gap of 1000 bp or a gap of the mean + 3 standard deviations (μ + 3σ) ≈ 652 bp for each library and chromosome was compared. The N50 scaffold size for scaffolds assembled using gaps < 1000 bp was 24.0 kb and for scaffolds assembled with gaps < (μ + 3σ) ≈ 584 bp was 18 kb, a difference of 33 %. For chromosome 12 the mean total length of scaffolds in each library for scaffolds assembled with gaps <1000 bp was 19 Mb (21 % of the length of BTA12) and for scaffolds with gaps < (μ + 3σ) was 15.8 Mb a difference of 20 %. The 20 % increase in total scaffold length when threshold gap length was increased by 71 % indicates that scaffold construction is relatively insensitive to the threshold gap length and hence that the precise choice of gap length is not critically important for scaffold construction. The subsequent SNP haplotype construction was performed with a fixed threshold of 1000 bp for joining scaffolds.

#### Effect of repeats on contigs and scaffolds

Contigs and scaffolds that fall within repeats could compromise the construction of SNP haplotypes since the reads could have been incorrectly mapped and repeat regions can give rise to false SNP calls. To discover whether contigs or scaffolds were affected by repeats, the numbers of contigs and scaffolds in, or overlapping, repeats were calculated for chromosome ten and compared with the number of contigs that would be within repeats if the same scaffolds were randomly placed (Table 1). There were 5.8 times as many scaffolds wholly within repeats in the observed data (31 % of scaffolds) as there were in the random data (5 % of scaffolds). The scaffolds that were wholly within a repeat were also about one fifth the length of other scaffolds (864 bp vs 4400 bp) in both random and observed data sets. The large excess of scaffolds wholly within repeats in the observed data suggests that many of these are mapping artifacts and that they should be excluded from the analysis. The excess of scaffolds in repeats was not reflected in an excess of contigs in repeats which was much lower (1.34 X) and therefore contigs in repeats were retained for scaffold construction but scaffolds wholly within repeats were removed from the subsequent analysis. Scaffolds in repeats tended to be short in both observed and random data and this is probably a consequence of the relatively short average length of repeats (487 bp).

**Table 1:** Observed and expected scaffolds in repeat regions. Counts and lengths of scaffolds with different interactions with repeats. There are 5.8 fold more scaffolds entirely within repeats than would be expected by chance

Classification	Observed		Random		Observed/Random	
	Count	Mean length^1^	Count	Mean length	Count ratio	Length ratio
Neither end in repeat	43,898	4373	75,834	3045	0.58	1.44
One end in repeat	78,390	4411	112,818	3321	0.69	1.33
Two ends in two different repeats	50,212	3686	47,842	3620	1.05	1.02
Whole scaffold within one repeat	77,348	864	13,414	814	5.77	1.06

^1^Mean scaffold lengths reported here are much shorter than the mean scaffold sizes reported in Table 4 from which scaffolds less than 1 kb have been removed

#### SNP data

Input files for the Single Individual Haplotyper (SIH) programme were prepared using BuildRefHapInput.V1.1.jar. Scaffolds were checked for excess heterozygous SNP before inclusion in the SIH input. Only loci that were heterozygous in both the bulk genomic DNA sequence data and had both alleles represented in the subcellular libraries were taken forward for SNP haplotype assembly. Before assembly, loci were tested against two quality criteria and loci failing these tests were excluded from the haplotype construction.

**Fig. 5:**
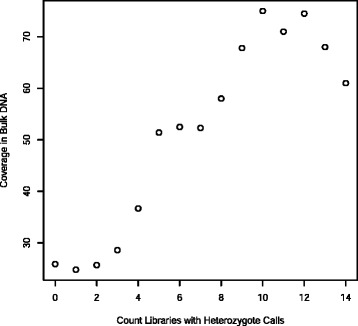
Coverage at SNP loci in bulk genomic DNA data plotted against the number of subcellular libraries in which heterozygotes were found. The strong correlation between depth of coverage in the bulk genomic DNA sample and number of heterozygote containing subcell libraries (r^2^ = 0.84) suggests that increasing copy number causes more libraries to contain contigs representing that region

If contigs were randomly placed across the genome and each subcellular library contained 1/6th of the cellular genome then it would be expected that about 9 % of the contigs would represent both orthologous chromosomes and might therefore contain true heterozygous SNP (1/6th of each chromosome is amplified therefore (1/6)^2^ = 2.8 % of the genome will be heterozygous but this is ~9 % of a sample that had gone into the library). For each subcellular library ~8 % of loci that were heterozygous in the bulk DNA were also heterozygous in that library; about the expected number. Apparent heterozygotes can also be a consequence of Copy Number Variants (CNV), repeats and sequencing errors. The coverage of all loci that were heterozygous in the sequence obtained from bulk genomic DNA was obtained and plotted against the number of libraries for which there was a heterozygote call at that position (Fig. 5). There was a strong correlation between coverage and the number of libraries that were detected as containing a heterozygous SNP at that position (r^2^ = 0.94). This would suggest that at least some of the heterozygous SNP calls were artefacts of CNV or expanded repeats in the tested animal or collapsed repeats in the reference genome assembly.

**Fig. 6:**
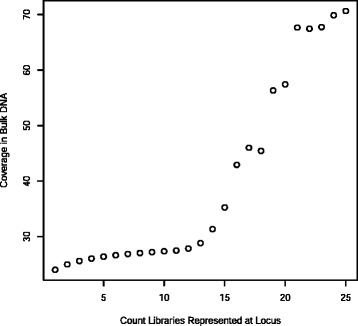
Association between coverage in the bulk DNA sample and number of subcellular libraries represented at the locus. Coverage is constant up to about 13 libraries at a locus. At loci with more than 13 libraries represented the coverage in the bulk genomic sample rises steeply suggesting that these loci are associated with CNV

There was a more complex relationship between the number of subcell libraries represented at a locus and the depth of coverage in the bulk genomic DNA sample. Coverage was between 24 and 28 fold up to about 13 libraries at a locus. Howevever at loci with more than 13 libraries represented the coverage in the bulk genomic sample rose steeply suggesting that these loci are associated with CNV (Fig. 6).

Up to six libraries were prepared from each cell, but only two contigs should be found covering any given genomic region in the sequence from a single cell. It is possible to obtain more than two copies if the region has a copy number greater than 1 per chromosome. Higher numbers of copies of a region would lead to more libraries containing contigs across the region and hence the observed association between numbers of libraries containing heterozygotes and coverage. Approximately 1.3 % of the Angus genome is associated with CNV > 20 kb and more is associated with smaller CNV [17]. In total, 46 % of the genome was classified as repetitive by Repeat Masker with a mean repeat size of 229 bp and a maximum of 8.5 kb. Some heterozygous SNP in subcellular libraries are therefore to be expected as a consequence of CNV and repeats and are not necessarily an indicator of both chromosomes being represented in a library. Scaffolds that contained more than three heterozygous loci or that had a heterozygote frequency greater than 0.2 were removed by BuildRefHapInput.V1.1.jar. These parameters were chosen after inspection of a small number of clearly mixed (heterozygous) scaffolds. This procedure removed 93 % of all heterozygotes in the data set and involved deleting 2.2 % of the scaffolds. This filter should remove scaffolds that genuinely are a consequence of both orthologous chromosomes being represented in the library and also scaffolds affected by repeats and CNV.

For any pair of adjacent SNP loci with alleles A/a and B/b there are 4 possible haplotypes (AB, ab, Ab, aB), only one or two of which should be present in any given diploid animal. The number of haplotypes was counted for all pairs of adjacent loci on chromosome 21 (Table 2). Four percent of the two SNP haplotypes had > 2 alleles but after filtering to remove both loci, over 90 % of the new two SNP haplotypes that spanned the original locus had two or fewer alleles leaving only 0.4 % of two SNP haplotypes with > 2 alleles. These residual anomalous loci were not further filtered. This procedure for removal of anomalous loci was applied to all chromosomes using BuildRefHapInput.V1.1.jar. The number of subcellular libraries at loci with > 2 two SNP haplotypes was compared with coverage. The coverage at loci with > 2 two SNP haplotypes increased directly with the number of subcellular libraries which contained these loci (*y* = 1.5× + c, r^2^ = 0.84). This suggests that these loci are caused by CNV, repeats or reference genome assembly errors rather than sample contamination.

**Table 2:** Counts of two SNP haplotypes. Counts of two SNP haplotypes with 1–4 alleles for chromosome 21 before and after filtering to remove the loci with more than two 2 SNP haplotypes. Removing any pair of adjacent loci creates a new two SNP haplotype. But the efficacy of the filtering is indicated by the 90 % reduction in loci with > 2 haplotypes after filtering

Allele Count	Count (%) loci before filtering	Count (%) loci after filtering
1	26,232 (40)	25,489 (45)
2	36,746 (56)	30,789 (55)
3	2830 (4)	214 (0)
4	18 (0)	0 (0)

#### Validation

Haplotypes were validated in three ways: first by testing for self-consistency of scaffolds; overlapping scaffolds from the 31 subcellular samples should either have all the same genotypes or all different genotypes. Second, scaffolds were tested for consistency with haplotypes constructed independently by Beagle using data from the Illumina 770 K BovineHD SNP chip. Third, haplotypes constructed using SIH which uses SNP to join scaffolds on the same haplotype were also compared against the 770 K SNP Beagle phased haplotypes.

For each approach two measures of consistency were obtained as previously described [3]. When comparing two scaffolds derived from the same animal all SNP alleles should be the same or all should be different since the scaffolds will be derived from the same or different haplotypes. The first measure of consistency was the greater of the percentage of positions that were identical or different in two scaffolds. However, if an error occurs during scaffold reconstruction then multiple contiguous alleles will be out of phase with the preceding alleles. Kaper et al. [3] described these as a “switch errors”; defined as two or more adjacent loci that were out of phase with the preceding loci. Switch consistency was calculated by disregarding switch errors and only counting single isolated loci that were out of phase with neighbours on both sides as an error. The effect on the two measures of consistency of using maximum gap sizes of 1000 to 5000 bp for joining contigs to form scaffolds was also examined. As the maximum gap sizes for joining contigs increases more switch errors are likely to occur and the difference between the two error rates can be used to identify an appropriate maximum gap length.

Table 3 shows the percentages of both types of errors for the three tests of consistency described above for the five maximum gap lengths. In addition, the consistencies obtained after using TargetCut to assemble scaffolds were also obtained. Self-consistency between scaffolds from different libraries was 99 % with a 1 kb gap length. As expected, self-consistency declined when using larger maximum gaps between contigs for scaffold construction but only to 98.3 % for a 5 kb maximum gap. Consistency when disregarding switch errors remained constant indicating that the decline in the uncorrected consistency score was due to inappropriate joining of contigs on different haplotypes. The uncorrected consistency of the scaffolds against the Illumina BovineHD SNP haplotypes was 92.8–96 % and 97 % for the corrected consistency. Finally, the uncorrected consistencies of SIH constructed haplotypes against the Illumina BovineHD SNP haplotypes were 89–96 % and after correction was ~97 %. Scaffolds obtained with TargetCut had consistencies similar to those obtained with gaps of 2 kb using ContigMetrics.

**Table 3:** Haplotype consistency after using five different maximum gap lengths for joining contigs into scaffolds. Haplotype consistency with and without corrections for switch errors for five different maximum gap lengths for joining contigs into scaffolds for chromosome one. The consistency values for all chromosomes with the default distance of 1000 is shown in the top line for comparison. The relative low value of consistency for the whole genome against SNP haplotypes (0.88) is due to three chromosomes with low consistencies (0.60–0.66), all other consistencies were > 0.87 and the median was 0.917. The consistencies obtained after using TargetCut to generate scaffolds is also shown. The N50 haplotype length obtained using SIH shows the large effect of increasing threshold gap length between contigs

	Against Other Scaffolds		Scaffolds Against HD SNP haplotypes		SIH phase haplotypes against HD SNP haplotypes		N50 SNP Haplotype Length (Mb)
Gap	Consistency	Switch Consistency	Consistency	Switch Consistency	Consistency	Switch Consistency	
All 1000	0.991	0.997	0.959	0.980	0.886	0.969	97
Chr1 1000	0.990	0.996	0.960	0.978	0.927	0.973	99
Chr1 2000	0.987	0.995	0.939	0.972	0.881	0.969	242
Chr1 3000	0.986	0.995	0.918	0.969	0.836	0.967	394
Chr1 4000	0.985	0.995	0.898	0.963	0.823	0.962	564
Chr1 5000	0.983	0.994	0.891	0.960	0.822	0.964	592
Chr1 TargetCut	0.988	0.995	0.942	0.971	0.894	0.969	223

Increasing the maximum gap length allowed for joining contigs into scaffolds increased the N50 scaffold length in almost direct proportion to the increase in gap length (Fig. 4) and N50 SNP haplotype length (Table 3). Since consistency declined relatively slowly with increasing gap size it is possible to use ContigMetrics with a threshold gap size that optimises the trade-off between haplotype size and accuracy for a particular application.

#### SNP haplotype construction

The size of the interval between informative SNP determines the resolution of haplotype boundaries. The mean interval between informative SNP in the sequence of the bulk genomic DNA sample was 1820 bp but the N50 distance was 9791 bp both of which are less than the mean (16 kb) and N50 (27 kb) scaffold lengths (Table 4). Therefore there are expected to be about nine SNP loci per scaffold and, although this will be very variable, there should be sufficient data to reconstruct haplotypes from SNP genotypes. Scaffolds less than 1000 bp were discarded since they were unlikely to contain two informative SNP loci and hence aid in SNP haplotype reconstruction (see below) and were also associated with repeats (see above).

**Table 4:** Descriptive statistics for scaffolds

Sample Set^1^	Mean Scaffold Length (kbp)	N50 Scaffold Length (kbp)	Max scaffold length (kbp)	Scaffold Coverage of Genome	% Scaffolds deleted for heterozygotes	% Consistency	% Consistency (Switch)	% Genome under (informative) Scaffolds	% Genes within Scaffolds	% Pairs Missense SNP phased
Angus Steer CM	16	27	421	8.1	2.2	99.10	99.70	98 (89)	65	99.96
Angus Steer TC	33	46	837	6.2	6.3	98.98	99.58	97 (93)	75	99.96
EAZ Cells CM	29	73	791	1.2	0.2	97.72	99.12	91 (56)	67	99.91
EAZ Cells TC	81	133	1711	1.7	1.7	95.98	98.55	89 (70)	75	99.91
EAZ Fosmids CM	26	36	791	11.5	3.3	97.57	99.51	99 (89)	76	99.97
EAZ Fosmids TC	43	41	1527	12.5	4.9	95.01	98.64	100 (95)	82	99.97
Angus Bull CM	55	80	1791	7.5	11.6	99.30	99.92	41 (41)	10	99.98
Angus Bull TC	180	413	52,056	6.4	5.5	95.38	98.87	96 (82)	70	99.98

^1^ *CM* = scaffolds constructed using ContigMetrics with a 1000 bp gap permitted between contigs; *TC* TargetCut in Samtools

After filtering out scaffolds with > 20 % or > 3 heterozygous loci and pairs of SNP loci with > 2 two SNP haplotypes, the remaining SNP loci were formatted for haplotype construction by SIH with input from scaffolds constructed using either ContigMetrics with a 1000 bp maximum gap or Samtools TargetCut. The mean and N50 size of scaffolds are shown in Tables 4 and the same data for SNP haplotypes compiled by SIH are shown in Table 5. Descriptive statistics are shown for the contigs (Table 6) and scaffolds (Table 4) for all sets of processed samples.

**Table 5:** Descriptive statistics for SNP containing haploid sequences constructed by SIH averaged over all autosomes

Sample Set^1^	Mean haplotype length (kbp)	N50 haplotype length (kbp)	Max haplotype length (kbp)	% SNP phased	% Genes Within haplotypes	% Genome in haplotype blocks
Angus Steer CM	43	97	738	88	38	64
Angus Steer TC	82	190	946	87	46	68
EAZ Cells CM	39	105	921	34	19	27
EAZ Cells TC	84	201	1653	30	42	32
EAZ Fosmids CM	65	134	1194	82	23	57
EAZ Fosmids TC	127	257	2457	80	48	66
Angus Bull CM	72	155	1243	31	12	22
Angus Bull TC	229	1142	7.878	53	14	38

^1^The low proportion of genome covered by the Angus Bull SNP haplotypes compared to the high genome coverage of scaffolds was partly due to a large proportion of scaffolds containing heterozygotes that were filtered prior to haplotype construction. For the Angus bull no scaffolds were used that contained heterozygotes, for the other sample sets scaffolds with a maximum of 3 heterozygotes or a maximum of 20 % heterozygotes were accepted and the heterozygous positions deleted

**Table 6:** Descriptive statistics for libraries and contigs

Libraries					Contigs				
Sample	Sequence type	Million reads /library	Gb mapped data/library	Count Libraries	Mean contig length (bp)	% Genome covered/library	N50 Contig length (bp)	Read depth over contigs	Contig coverage of genome
Angus Steer	SOLiD 1 x 75 bp	34.5	2.6	31	165	14.2	359	7.7	4.2
EAZ Cells	HiSeq 2 × 100 bp	39.2	2.6	21	796	5.6	3369	32.9	1.9
EAZ Fosmids	HiSeq 2 × 100 bp	28.5	2	47	1288	21.8	3209	4.6	10.7
Angus Bull	SOLiD 1 × 75 bp	166.01^1^	9.1	8	198	50	479	5.6	4

^1^The high number of reads for the Angus bull was in part due to a single outlier library, after excluding that library the number of reads was 88 M

The N50 scaffold length for the Angus steer was 27.4 kb and the N50 haplotype length for SNP based haplotypes obtained by SIH was 97 kb. 89 % of the genome had scaffold coverage of at least 1 X but only 64 % of the genome had haplotypes assigned by SIH. This may at least in part be due to the distribution of gaps between informative SNP. Although 50 % of the genome had SNP less than 2446 bp apart, 22 % had SNP > 10 kb apart and 15 % > 20 kb apart. Haplotypes in these regions might not be captured by the scaffolds, which had mean and N50 lengths of 16 kb and 27 kb respectively. Although <90 % of the genome was covered by informative scaffolds or SNP haplotypes > 99.96 % of heterozygous SNP loci were phased on scaffolds and 88 % on the longer SNP Haplotypes, supporting the hypothesis that <90 % of the genome was covered by informative scaffolds or SNP haplotypes because of an absence of informative SNP in these regions.

#### Comparison of sample sets

Libraries were prepared from four sets of samples. Genomiphi amplified single cells from: 1) an Angus steer prepared from freshly collected cells at the University of Missouri; 2) single sperm cells from an Angus bull prepared from semen shipped at ambient temperature from Missouri USA to Liverpool UK; 3) blood of EAZ cattle collected in Western Kenya and stored at ambient temperature (20–38 °C) for 24–48 h prior to amplification. 4) a fosmid library from bulk genomic DNA of a single EAZ cow without Genomiphi amplification.

Furthermore two different sequencing technologies were used. The Angus samples were sequenced from 75 bp fragment libraries on Applied Biosystems SOLiD 5500 machines and the EAZ samples were sequenced from 2 × 100 bp paired-end libraries on an Illumina HiSeq.

The fosmid library was supplied in pools of 6000 fosmids with 30–45 kb inserts and three pools were combined for each library preparation. Each library was expected to cover 24 % of the haploid genome and 6 % of the sequence data in a given library was expected to come from both orthologous chromosomes ((0.24/2)^2^)/0.24). Sequence from each library covered approximately 22 % (σ 4.5 %) of the haploid genome suggesting that the stated fosmid pool size was accurate.

The contig and scaffold length parameters reflected the different sources of the libraries and the different sequence data types and analysis methods.

**Fig. 7:**
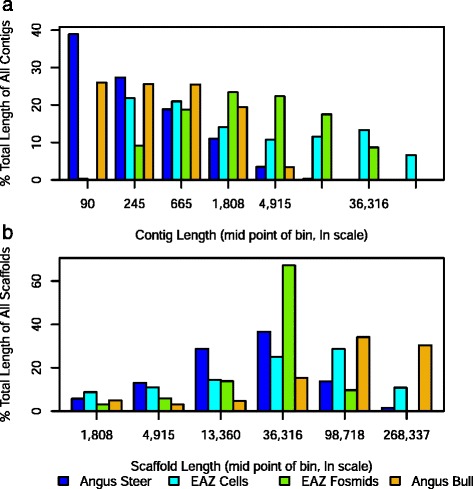
Distributions of contig lengths (Panel **a**) and scaffold lengths (Panel **b**) for the four sets of samples. The two 75 bp fragment libraries from the Angus cattle had much shorter contigs than did the 2 × 100 bp paired-end libraries from the EAZ animal

Effect of sequence data type: The 2 × 100 bp paired-end libraries sequenced on the Illumina HiSeq generated contigs at least four times longer than the 75 bp fragment libraries sequenced on the ABI SOLiD. However, the scaffold lengths from the comparable Angus Steer (SOLiD) and EAZ Cells (HiSeq) only differed by two to three fold. Contig lengths of the two 75 bp fragment libraries from Angus animals had similar distributions (Fig. 7). More of the total lengths of contigs from the EAZ cells were in short contigs than the contigs from EAZ fosmids presumably because of the low coverage of this sample.

Effect of sample source: We sequenced DNA from aliquots of single cells, single intact sperm cells and a fosmid library. The unfragmented sperm cells generated the longest scaffolds as expected (N50 scaffold length of 80 kb with ContigMetrics and 413 kb with TargetCut). Given that the sperm were shipped from the USA to UK at ambient temperature (within a preservative buffer), it is also an indicator of the robustness of these cells.

The fosmid library generated scaffolds with N50 scaffold sizes that approximated to the expected insert size (~40 kb) and these had the smallest variability in scaffold size (standard deviation) relative to mean scaffold length (Fig. 7). The N50 sizes of scaffolds found by ContigMetrics and TargetCut were similar. TargetCut was developed for fosmid samples and generated a mean and N50 scaffold length that only differed by 5 % (Table 4), which also indicates that the length distribution was almost symmetrical. Sixty five percent of the total scaffold lengths from the EAZ fosmids were in the 22–60 kb range whilst scaffolds from other libraries had a broader distribution (Fig. 7). The scaffold length of the libraries from subcellular aliquots (Angus steer and EAZ cells) had N50 lengths between 16 kb and 51 kb depending on source and analysis method and the N50 scaffold lengths were larger than the mean scaffold lengths, indicative of a skewed distribution with many shorter scaffolds. Given the good approximation of the fosmid scaffolds to the expected fosmid sizes, the scaffold sizes derived from the subcellular fragments may also reflect the size of the chromosome fragments or the phi29 polymerase products derived from them. The sperm cells from the Angus bull had the largest proportion of the scaffold lengths in long scaffolds. This is presumably because the sperm cells were not aliquoted and hence there was no opportunity for fragmentation by pipetting (Fig. 7).

The importance of the sample source is further emphasised by the large variation in the amount of available data, which varied between 1.2 X and 12.5 X scaffold coverage. The scaffold coverage of the genome from the EAZ subcellular libraries was particularly sparse at 1.2 X. But there was no correlation between scaffold coverage of the genome and N50 scaffold length (r^2^ = 0.002), supporting the hypothesis that scaffold lengths represent the lengths of the underlying fragments.

Effect of scaffold reconstruction method: TargetCut was specifically designed for reconstructing scaffolds of reads across fosmids and assumes that scaffolds will be in the range 25–45 kb; it appears to have been very successful at obtaining scaffolds with both mean and N50 sizes close to the expected size (~40 kb) for the fosmid library. However, where the template does not conform to this expectation, TargetCut risks joining contigs from adjacent shorter scaffolds. The mean lengths of scaffolds generated by TargetCut on subcellular fractions were 2–3 times longer than those generated with ContigMetrics when using a maximum permitted gap between contigs of 1000 bp. ContigMetrics with a gap of 2000 bp generated similar SNP haplotype lengths to TargetCut (Table 3) but preliminary analysis suggested that gaps in the 1–2000 bp range were randomly distributed, which provides no support for combining such distant contigs into scaffolds. There was a modest loss of data and data quality to offset this substantial gain in scaffold length when using TargetCut; consistency was ~2 % greater with ContigMetrics and the number of scaffolds that had to be rejected because they contained excess heterozygotes was 1–4 % greater with TargetCut, with the exception of the sperm data, which was an outlier in many respects. The choice of scaffold assembly method could be guided by the relative importance of size of scaffold and correctness of assembly.

A key objective of haplotype reconstruction is to identify the phase of pairs of functional alleles within a gene. In order to test the sensitivity of the sequence-based haplotyping we estimated the percentage of pairs of non-synonymous (missense) SNP that would be on the same scaffold or SIH SNP based haplotype. Over 99 % of pairs of missense SNP within a given gene shared at least one scaffold (Table 4) suggesting that the majority of potentially functional interactions between polymorphisms in genes will be captured by this method.

The mean length of cattle genes in Ensembl79 was 32 kb (N50 length 106 kb) which is substantially greater than the 10 kb mean haplotype length estimated by linkage disequilibrium [18]. The very long genes (>100 kb) require correspondingly very long scaffolds or haplotypes to cover them and many were not fully covered. Tables 4 and 5 show the percentages of genes entirely with a single scaffold or SNP haplotype respectively. For most samples and analyses 65–82 % of genes were within scaffolds but only 19–48 % were within SNP haplotypes despite the greater length of SNP haplotypes. This is likely to be because multiple scaffolds but only one or two SNP haplotypes cover any given locus.

Annotation: SNP in genes were annotated with their consequences for gene structure using the Ensembl API. Counts of each type of structural change are shown in Table 7. The EAZ have many more SNP in all categories. This is probably for two reasons: first, the EAZ cell samples were collected from 18 different animals and not just one. Second, EAZ cattle are primarily of Bos indicus origin with some Bos taurus introgression, whereas the reference sequence and the Angus cattle are of Bos taurus origin. Bos taurus and Bos indicus cattle separated about 200,000 years ago and have adapted to different environments as well as accumulating neutral variants.

**Table 7:** Counts of consequences of SNP for gene structure. Counts of consequences for gene structure of SNP within genes for each group of sequences

Consequence	EAZ	Angus Cow	Angus Bull
3 prime UTR variant	25,922	8709	8272
5 prime UTR variant	4773	1255	1259
Coding sequence variant	134	26	16
Downstream gene variant	100,961	32,248	27,382
Initiator codon variant	84	32	31
Intron variant	3,672,920	1,250,026	1,077,417
Mature miRNA variant	70	39	40
Missense variant	37,979	14,081	14,774
Non-coding transcript variant	6027	2258	1931
Non-coding exon variant	5749	2186	1872
Splice acceptor variant	252	97	195
Splice donor variant	251	92	144
Splice region variant	9735	3136	3120
Stop gained	401	196	403
Stop lost	23	8	16
Stop retained variant	37	8	11
Synonymous variant	53,102	16,231	12,837
Upstream gene variant	91,273	30,948	27,759

The counts of non-synonymous (missense) and synonymous variants were obtained for each gene in the EAZ cattle. The 414 genes with a ratio of non-synonymous/synonymous > 4 were submitted to DAVID to identify pathways that might be under strong positive selection. The only cluster of genes with an enrichment score > 1 contained 29 olfactory receptor genes (Enrichment score 4.29).

We have demonstrated a simple method for obtaining direct molecular haploid sequences by isolating and amplifying fractions of individual cells. Molecular haplotypes have been obtained before, by a range of methods [3, 4, 11, 14]. The key objective of our protocol was to reduce the complexity and cost of the procedure in order to make it available to any laboratory with access to next generation sequencing. Our method only requires a haemocytometer and a microscope and we minimised the number of libraries to be sequenced and the amount of sequence obtained from each library. The amount of raw sequence data obtained for the Angus steer was in the same range (30 X genome coverage) as would commonly be used for a high quality consensus sequence. Making 30 libraries instead of just one adds to the cost of haploid sequencing and this is true of all methods proposed to date. Kaper et al. [3] and Kuleshov et al. [4] have also obtained haploid sequences by limiting dilution. Kaper et al. [3] diluted bulk genomic DNA and used phi29 polymerase to amplify the aliquots. Kuleshov et al. [4] sheared bulk genomic DNA to 10 kb fragments, aliquoted the fragments into pools of 3000–6000 fragments and amplified these by long range PCR. This latter strategy should produce a less skewed read coverage distribution than phi29 polymerase, but may suffer more problems with hard to amplify regions. Alternative methods of amplifying DNA from trace samples such as MALBAC [19] should also generate less skewed coverage than phi29 amplification and be capable of amplifying from longer template fragments than long range PCR.

Kaper et al. [3] used 96–192 libraries per sample and Kuleshov et al. [4] used 384–768 libraries per sample. In these protocols the cost of library construction can exceed the cost of sequencing. Reagents for library preparation from the sequencer manufacturers cost ~ $50–$100 per library in the UK and reagents for 100 Gb of sequence to give ~ 30 X genome coverage costs ~ $6000. The number of required libraries depends on the desired level of scaffold coverage but also on the fraction of the haploid genome in each sample. We used 0.33 haploid equivalents and Kaper et al. [3] used 0.4 haploid equivalents per sample, however, Kuleshov et al. [4] used 0.015 haploid equivalents. Using smaller fractions of the genome reduces the risk of sequencing template derived from both orthologous chromosomes but increases the number of libraries required. We expected about 9 % of scaffolds to be derived from both chromosomes and the value for Kaper et al. [3] was similar but for Kuleshov et al. [4] the fraction would be about 5 × 10^−5^. If scaffolds from both chromosomes can reliably be identified and filtered out then an optimal sequencing strategy would minimise the combined cost of sequencing and library construction. By removing 2.2 % of scaffolds from the data set we eliminated 82 % of heterozygous SNP in scaffolds. The majority of the remaining heterozygotes were assumed to be due to CNV and repeats. A cost calculator for different strategies is included in the Supplementary files (CostCalculator.xls). The project cost varies only by about 10 % for dilutions between 0.5 and 0.1 genome equivalents per library, but using a very small proportion of the genome, as did Kuleshov et al. [4], increases project costs almost threefold at manufacturer’s current list prices. At > 0.1 genome equivalents per library the main determinants of cost were the target depth of read coverage over scaffolds and the scaffold coverage of the genome. Read coverage of scaffolds has to be sufficiently high to detect a large proportion of heterozygotes within a scaffold to mark it for removal. If read coverage is uniform then 4 X read coverage of scaffolds would detect 61 % of heterozygotes which should be sufficient to detect most diploid scaffolds (Supplementary data CostCalculator.xls). However genome amplification with phi29 is very skewed so scaffold coverage would need to be correspondingly increased; with long PCR [4] or MALBAC [19] 4 X coverage should be adequate. With very high genome dilutions then detection of heterozygotes becomes unnecessary and coverage can be correspondingly lower, Kuleshov et al. obtained consistent and long haplotypes with a 2 X scaffold coverage.

There are no established quality criteria benchmarks for a diploid genome sequence and criteria will ultimately depend on the intended application. For understanding long-range interactions within the genome of a given individual haplotypes of 1 Mb or greater could be desirable, for example for resolving HLA (or BoLA) haplotypes. However for understanding the haplotype structure of populations, to increase the power of mapping studies, then much shorter haplotypes that capture most SNP in linkage disequilibrium within genomic regions harboring quantitative trait loci would be sufficient. We used simulation to obtain the percentage of haplotypes that would be completely covered by at least one scaffold given ratios of scaffold length to population haplotype length of between 1 and 7 and scaffold coverage of 2–20 X, assuming haplotype lengths are exponentially distributed and that the mean haplotype length in the cattle that we sequenced was 10 kb [18] (Table 8). With the coverage that we achieved we would expect to capture ~99.9 % of the Angus steer population haplotypes within scaffolds; 82 % with the EAZ cells; 99.9 % EAZ fosmids and ~100 % Angus sperm, all with the TargetCut analysis but ContigMetrics gave similar results except for EAZ cells where coverage was predicted to achieve 70 %. Since less than 100 % of the genome was covered by scaffolds, these percentages need to be adjusted for the actual percentage of the genome covered (97.5–99.9 %).

**Table 8:** Percentage of population haplotypes expected to be covered by a scaffold for different mean scaffold lengths and scaffold coverage of the genome

	Scaffold coverage									
Mean scaffold length (bp)	2	4	6	8	10	12	14	16	18	20
10,000	25	45	56	63	67	72	76	78	80	81
20,000	42	67	80	87	91	94	95	96	97	97
30,000	49	77	90	95	96	98	99	99	99	100
50,000	56	86	95	98	99	100	100	100	100	100
70,000	62	89	97	99	100	100	100	100	100	100

SIH uses the SNP data from all scaffolds to form a single pair of SNP haplotypes from all overlapping scaffolds. The SNP haplotypes are expected to be larger than the scaffolds and this was found, although mean SNP haplotype lengths were only marginally longer than mean scaffold lengths for the EAZ cells, presumably because these cells were drawn from a population not a single animal and therefore overlapping scaffolds would not necessarily be consistent. Although N50 lengths for SNP haplotypes were large (97–190 kb) for the Angus steer there was a substantial loss of data since genome coverage fell from ~ 91 % for the informative scaffolds to ~66 % for the SNP haplotypes. The ability of any method to reconstruct whole genome haplotypes will ultimately be determined by the length of scaffolds used and the lengths of homozygous regions across the genome. Homozygous regions might be quite short in highly outbred populations, such as many human groups, but correspondingly large in intensively selected livestock such as the Angus cattle used here. The mean and N50 gap between informative SNP used for haplotype reconstruction was 1820 bp and 9791 bp. The maximum gap between informative SNP was 1.08 Mb on BTA7.

The N50 length of scaffolds in the Angus steer was 46 kb, which is substantially longer than the 14 kb N50 scaffolds obtained by Kaper et al. [3] using dilutions of bulk DNA and the same analysis method (TargetCut). This suggests that the direct dilution of DNA may have fragmented the template more than taking aliquots from individual cells. The N50 SNP haplotype length obtained using SIH by Kaper et al. [3] was dependent on the number of libraries and depth of sequence, ranging from 197 kb N50 haplotype length for 96 libraries and 29 X genome coverage to 702 kb N50 haplotype length from 192 libraries at 88 X genome coverage. We observed a N50 haplotype length of 190 kb for the Angus Steer when using TargetCut with 31 libraries and 29 X genome coverage, respectively. Despite using a similar coverage and less than a third of the number of libraries that Kaper et al. [3] used we obtained haplotypes that were 96 % of the length, which may well be attributable to the longer scaffold length obtained from diluted cells rather than diluted DNA. The relative efficiency of our method may be greater than these data suggest since we were working with inbred cattle with long homozygous regions that could not be linked by informative SNP. Therefore the use of cell aliquots rather than diluted DNA seems to generate data of similar quality with only one third of the libraries and increases the ease of library preparation (Kaper et al. [3] state that preparing accurate dilutions is “challenging”).

Molecular haplotypes have also previously been obtained by fosmid cloning and TargetCut and SIH for haplotype reconstruction, which produced N50 haplotypes of 1 Mb and 81 % of genes completely within haplotypes[14]. This is about five times larger than the N50 of the haplotypes that we obtained using 75 bp fragment reads on the Angus steer and four times larger haplotypes than we obtained using fosmid libraries. However Suk et al. used more libraries (67 v 31 and 67 v 47) and more data (142 Gb vs 83 Gb and 142 Gb vs 108 Gb) than did we for the Angus steer and EAZ fosmid sequences, respectively. Furthermore, our cattle samples were likely to be more inbred than the human samples used by Suk et al. The number of libraries and amount of sequence obtained by either method can be adapted to the particular objectives of the experiment. The N50 haplotype length that we obtained from fosmids was 257 kb and >99 % of haplotypes were longer than 20 kb. This is sufficient for many purposes since > 95 % of human SNP are < ~10 kb apart [10, 20] and therefore the data presented here should be sufficient to tag most haplotypes.

## Conclusion

We have demonstrated a simple sample preparation method for generating DNA derived from single chromosomes. This together with the suite of data processing tools we have written will enable staff in any laboratory to obtain direct molecular haplotypes. The N50 size of scaffolds of paired end Illumina reads assembled with targetcut on the EAZ cells were 133 kb and covered 89 % of the genome. Using SIH to infer SNP haplotypes extended the N50 size in the EAZ cells to 201 kb. These diploid sequences of the EAZ cells were obtained using a genome read coverage of 19.5 X, similar to a standard high quality resequencing project. The preparation of 21 libraries rather than a single library was the only significant source of increased costs, and this cost penalty is likely to decline with the maturation of laboratory automation and library preparation kits.

This strategy therefore puts direct haplotype sequencing of diploid genomes within the reach of any laboratory. The method may be particularly useful for haplotype sequencing of large numbers of samples around loci of interest, since amplified aliquots of single cells can be screened by PCR to identify those containing the locus. This provides a route to sequencing megabase regions without complex cloning or primer walking or the discovery biases inherent in sequence capture.

## Methods

### Ethics

The project followed UK, US and Kenyan Institutional and Government Guidelines for Experiments involving animals. The only samples used were blood and semen samples. Blood collection from cattle is not a regulated procedure in the UK and a Home Office licence is not required for its collection. Blood samples collected in Kenya and the US were aliquots of larger samples that were collected for other purposes and also did not require ethical approval. The semen sample was from a US semen bank.

### Workflow outline

**Fig. 8:**
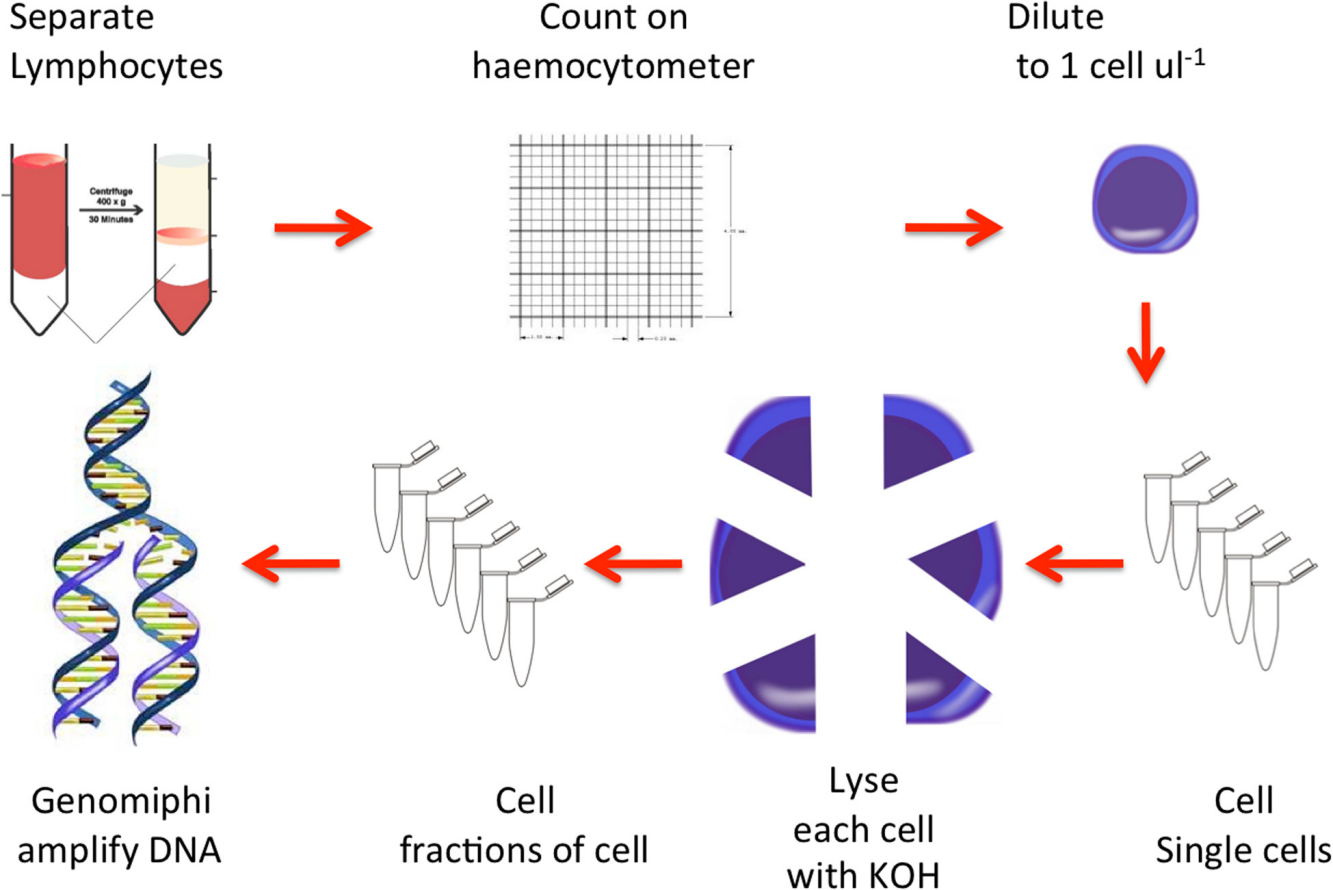
Sample processing workflow. Lymphocytes are prepared from whole blood and are then diluted with the use of a haemocytometer to count the concentration of cells at each dilution. Aliquots containing single cells were taken and lysed and six aliquots were taken from each single cell sample and independently amplified using Genomiphi

A diagram of the sample processing workflow is shown in Fig. 8. Lymphocytes were diluted to a concentration of 1 cell per microlitre by serial dilution and counting was performed on a Fuchs-Rosenthal haemocytometer. One μl aliquots were collected, lysed then neutralised and the lysate divided into six aliquots to make samples that would each be expected to contain 1/6th of the DNA from a single cell. Each aliquot was whole genome amplified with Genomiphi™ (containing the phi29 polymerase) (GE Healthcare Life Sciences, Piscataway, USA) before library preparation for short read sequencing.

### Samples

Samples were collected from two sets of animals. First, from a single Angus steer (number 72711) from the University of Missouri research farm and a semen sample from a US registered Angus bull (Lab Id 1736). Second, from 18 East African Zebu (EAZ) cattle sampled in Busia district near Lake Victoria in Western Kenya in January and February of 2012. Finally, a fosmid library was prepared from EAZ cow 121-147 collected from Busia at the same time.

### Preparation of cells

Ten milliliters blood samples were collected into vacutainers with acid citrate dextrose anticoagulant and processed within two hours in the case of the Angus steer. In the case of the EAZ, blood samples were stored at ambient temperature (22–37 °C) for up to 48 h. Lymphocytes were separated from whole blood using Lymphoprep™ (STEMCELL Technologies, Vancouver, Canada) following the manufacturer’s instructions. As well as eliminating red blood cells Lymphoprep also eliminates neutrophils, which are the leukocytes with the shortest half-life (~4 h). Residual red blood cells were removed by ammonium chloride lysis (ACK, Lonza, Basel, Switzerland) and the remaining lymphocytes were resuspended in phosphate buffered saline (PBS).

A semen sample from Angus bull 1736 was used in the dilution procedure below without prior preparation.

### Cell dilution and amplification

Cells were counted on a Fuchs-Rosenthal haemocytometer using the entire area within the triple grid lines; the volume over this area is 3.2 μl. Cells were serially diluted in PBS and at each dilution an aliquot was taken and stained with 1 % Lugol’s iodine for visualisation. This procedure was repeated until an average of ~3 cells were observed in the whole area of the haemocytometer over five replicate counts of the same dilution. Whole genome amplification of single chromosomes was adapted from the detailed protocol of Spits et al. [3, 21] for whole genome amplification from single cells. One μl aliquots of the final cell dilution were immediately added to 1.5 μl of alkaline lysis buffer (KOH 200 mM, DTT 50 mM). Aliquots were frozen at−80 °C for at least 30 min and for up to 6 days, incubated at 65 °C for 10 min, chilled on ice and briefly centrifuged to collect the condensate. To each 2.5 μl of lysate 9.5 μl of Genomiphi^TM^ (Invitrogen) sample buffer was added and mixed by pipetting to neutralise the sample. Two μl of the neutralised lysate was used in each 20 μl Genomiphi^TM^ reaction following the manufacturer’s protocol. The resulting product was called a subcell sample. The remaining 10 μl of lysate was stored at −20 °C. Genomiphi products were visualized by electrophoresis in 1.5 % agarose gels. For samples that generated detectable Genomiphi amplification products, the remaining 10 μl of lysate was used in 5 further Genomiphi reactions to make a total of six Genomiphi subcell products per cell.

A second Genomiphi^TM^ reaction was performed using 20 ng of the first Genomiphi^TM^ product to generate material for validation purposes. Each of the six subcell products from a single cell was expected to contain sequences from approximately 1/3rd of the haploid genomes (1/6th of the diploid genome). Each subcell product was tested with 24 PCR primers spaced across the bovine genome. The perfect subcell product is expected to yield ~8 positive reactions (2 × 1/6^th^ × 24). Subcell products that had less than 15 positive PCR reactions were judged to be from a single cell and were selected for short read library preparation. The semen sample was diluted in the same way and aliquots predicted to contain whole single sperm were amplified and processed as described above. Whole genomic DNA was prepared from bulk cells from the Angus animals using the DNeasy Blood & Tissue Kit (Qiagen, Hilden, Germany). A fosmid library with 35–45 kb inserts was prepared by LGC Genomics (Berlin, Germany) from bulk DNA prepared from cells from a single EAZ cow that were stored and shipped frozen in 70 % ethanol.

### Sequencing

A barcoded library was prepared from each sperm or subcell aliquot of the Angus animals using the ABI SOLiD4 fragment library kit following the manufacturer’s instructions (Life Technologies, Foster City, USA). Fragment reads of 75 bp were obtained using ABI 5500 instruments and the exact call chemistry (ECC) module. ECC makes it possible to convert the color space data to nucleotide base sequences prior to mapping.

A separate library was prepared from DNA extracted from 50 cells from the Angus steer, which was amplified with Genomiphi and sequenced on the ABI SOLiD 5500 (Life Technologies, Foster City, USA). A library was also prepared from whole genomic DNA of the Angus steer using the Illumina PE-102–1001 paired end library kit and 2×100 bp reads were obtained with an Illumina GAIIx instrument (Illumina, San Diego, USA).

Libraries from the EAZ samples were prepared using the Illumina TrueSeq protocol and 2 × 100 bp reads were obtained on an Illumina HiSeq2000.

The fosmid library was aliquoted into 192 pools each predicted to contain ~6000 unique clones. Three pools of 6000 clones were merged to make each of 47 libraries using the Illumina TruSeq Paired End library preparation kit and 2 × 100 bp paired end sequence reads were obtained on an Illumina HiSeq2000.

### Data analysis

**Fig. 9:**
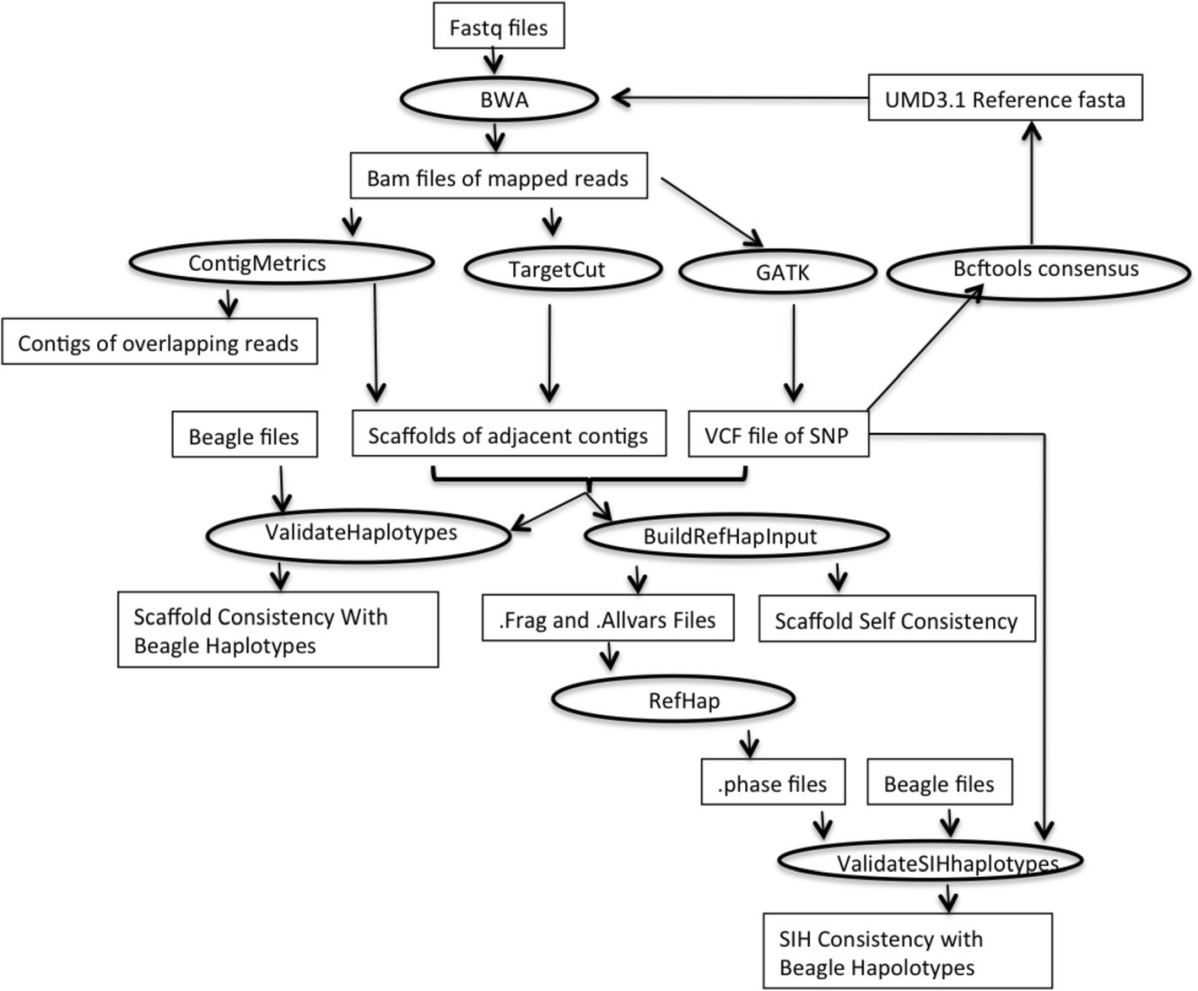
Data processing workflow. Fastq files are mapped to a reference with BWA to generate BAM files of aligned reads. ContigMetrics and TargetCut are used to generate scaffolds from the BAM files. GATK is used to generate VCF files. BuildRefHapinput takes VCF files and lists of scaffolds and outputs scaffold self consistency metrics and the input files for RefHap which is used to generate files of phased SNP using the SIH algorithm. ValidateSIHhaplotypes and validate haplotypes take haplotypes derived from SNP genotype data produced, e.g., with Beagle and compares them with haplotypes obtained by SIH and derived from scaffolds respectively

A diagram of the data processing workflow is shown in Fig. 9. A description of software written for the project and deposited at GitHub is included in Supplementary Files “Data Analysis Pipeline”. Contigs and scaffolds were constructed from the reads by mapping to the bovine reference genome assembly (UMD3.1 [22] GenBank GCF_000003055.4). Scaffolds that contained > 20 % or > 3 heterozygotes were deemed to come from both orthologous chromosomes and were removed. Pairs of adjacent SNP loci that had more than two alleles were deemed to have been generated by poor mapping due to CNV or repeats and the loci were removed but the scaffolds were retained. SNP data were then used to construct haplotypes from the scaffolds using the SIH software [13, 14] to join scaffolds that had the same allele at overlapping loci.

Programmes were written in Java to build scaffolds, filter out poor quality scaffolds and sites, format the data for SIH and validate the data by internal consistency and consistency with Illumina SNP chip and Beagle 3.3.2 derived haplotypes. To avoid excess memory usage and to take advantage of the available cluster all data processing was performed in parallel on a chromosome by chromosome basis leading to > 3000 calls to each programme to analyse data from each of the ~100 libraries across each of the 30 bovine chromosomes. Perl scripts were written to manage the calls to established and new software and undertake minor housekeeping tasks. A complete workflow together with scripts and source code for Java programmes are available on GitHub [4, 23].

### Mapping

ABI SOLiD reads were converted from color space to FASTQ base-space files using the ABI tool ConvertFromXSQ.sh with the -f switch, which removes reads which fail the ABI quality criteria. Illumina FASTQ files were directly processed. Reads were mapped to the UMD3.1 bovine reference genome [22] using BWA [24]. Reads were trimmed for quality using the-q 20 option, which is not equivalent to trimming bases with base quality < 20. Using-q 20 was found to increase the amount of mapped data from 53 to 73 %. The BWA-q algorithm is described in Supplementary data “Data Analysis Pipeline.” All other parameters controlling mapping were left at default values. GATK was used to call SNP (see below) and a new reference was created using bcftools consensus with IUPAC redundancy codes for heterozygous positions and the alternate allele for homozygous alternate alleles.

### Read scaffold construction

A Picard Tools module “ContigMetrics” was written to take BAM files from each library and build contigs of overlapping reads and then merge adjacent contigs that are within a user defined distance into scaffolds and output gff3 files with the co-ordinates of read contigs and scaffolds for each chromosome and library and also to report basic statistics for read and contig coverage.

It has been observed that phi29 amplification from trace amounts of template gives rise to a highly skewed distribution of read coverage with some genomic regions having very high coverage and others having little or none [7]. Therefore, given the low mean coverage of many libraries (1.6–40 X coverage of contigs), it was expected that even where template was present it was likely that there would be gaps in read coverage. To compensate for this, two methods were tested for joining adjacent contigs into scaffolds and these were implemented in the ContigMetrics module. First, the distribution of gaps between contigs at different scales was examined using histograms and fixed threshold distances were chosen for joining adjacent contigs. Second, the standard deviation of the gap length between contigs was obtained and different numbers of standard deviations were tested for joining adjacent contigs.

TargetCut also generates scaffolds from BAM files; it is available within Samtools and was developed for building scaffolds from sequences from fosmid libraries: “inserts were identified by computing read depth genome-wide in 1 kb windows for each clone pool and selecting runs of 25 to 45 kb for which at least two-thirds of the constituent windows had read depth above the predicted background level” [25]. This strategy makes assumptions about insert length that are probably not applicable to libraries prepared from DNA amplified from single molecules but it does appear to have been successfully used by others for this purpose [3]. We also used TargetCut for scaffold assembly to allow the direct comparison of our haplotype metrics with those of others.

### SNP calling

SNP were called using the Genome Analysis Tool Kit V2.4.7 [26] and Picard Tools [27] following the GATK Best Practice Guidelines (http://www.broadinstitute.org/gatk/). Duplicate reads were removed using Picard Tools and reads were twice realigned around indels, using GATK before calling SNP with the UnifiedGenotyper. GATK was run in DISCOVERY mode with EMIT_ALL_CONFIDENT_SITES set to ensure that reference bases were called where data were available to support them. Positions with read coverage greater than 50 were ignored. All other parameters were left at default, which meant that SNP required a minimum Phred scaled score of 30 to be called as high confidence, but low confidence SNP were also recorded.

### Data quality control and SNP haplotype reconstruction

A Java Programme BuildRefHapInput.V1.1.jar was written to process gff3 files of scaffold co-ordinates and Variant Call Format (VCF) files of SNP and output the frag and allvars files for processing by the SIH programme [14].

BuildRefHapInput.V1.1.jar filtered out all positions in the VCF file that were not informative amongst the processed libraries since these disrupted the consistency calculations. It also screened all scaffolds for an excess of heterozygous SNP that could be indicative of CNV or multiple chromosomes being represented in the sample. Scaffolds that contained more than 3 heterozygous loci or that had a heterozygote frequency greater than 0.2 were removed. These parameters are user defined and were chosen after inspection of a small number of clearly heterozygous scaffolds. BuildRefHapInput also compared all overlapping scaffolds from different libraries and calculated two measures of consistency between independent libraries as previously described [3]. The raw percentage consistency was the higher of the percentage of allele calls that were the same or the calls that were different. The switch distance is a similar calculation but assumes that errors will appear independently and that if two adjacent loci are inconsistent with the two preceding loci then it is unlikely to be sequencing error and that a haplotype switch has occurred and these errors are disregarded. Finally, the program generated the frag and allvars input files for the SIH programme for building SNP haplotypes from diploid sequence data [12, 13]. SIH was then used for haplotype reconstruction.

### Data validation

Scaffold haplotypes and SIH generated haplotypes from the Angus steer were compared against haplotypes inferred by Beagle 3.3.2 [12] from the Illumina BovineHD 770 K SNP genotype data from the same animal simultaneously phased in 3570 Angus individuals. Scaffold and SIH based haplotypes were validated using ValidateHaplotypes.jar and ValidateSIHHaplotypes.jar, respectively. The same consistency metrics were obtained for SNP haplotypes as described above for consistency between scaffolds.

### Percentage of haplotypes sequenced

A Java programme SimulateScaffoldCoverageHaplotypes.jar was written to simulate the exponential distribution of haplotypes across a chromosome in order to estimate the percentage of haplotypes in cattle that were completely covered by sequence haplotypes. Population haplotype lengths were assumed to be exponentially distributed with a mean of 10 kb [18]. Scaffold lengths were also assumed to be exponentially distributed. Haplotypes with exponentially distributed random lengths with a mean of 10 kb were tiled across an 86 Mb chromosome (the mean autosomal chromosome size for cattle). Scaffolds with exponentially distributed random lengths with mean obtained from Table 4 were placed randomly on the chromosome to a scaffold coverage depth also from Table 4. The scaffolds were then searched for those that would completely cover each haplotype on the chromosome. The length of haplotypes that were not completely covered by at least one scaffold was recorded and the percentage of the chromosome in haplotypes completely covered by scaffolds was reported. Table 8 shows the output for a range of depths of coverage and mean scaffold lengths in order to assist in experimental design.

### Supporting information

1. Data analysis pipeline; describes programmes to process the sequence data and their usage.
2. CostCalculator.xls: Can be used to explore the trade-off between numbers of libraries and proportion of genome in each library and depth of coverage.

### Availability of data and materials

Fastq files for all reads are deposited at the European Nucleotide Archive under SUBMISSION_ID ERA303977; STUDY_ID ERP005648.

SNP for each project will available from dbSNP with submitter handle CGR_LIV (http://www.ncbi.nlm.nih.gov/projects/SNP/snp_viewTable.cgi?handle=CGR_LIV).

Scripts and programmes are available from Github together with the Java source code https://github.com/LiverpoolHarry/HapSeq

Scaffolds and SNP haplotypes are available from our website http://www.genomics.liv.ac.uk/tryps/HaploSeq.html together with links to all other data.

## Abbreviations

ACK: Ammonium chloride potassium
BAM: Binary sequence alignment/map
BoLA: Bovine leukocyte antigen
BTA: Bos taurus
BWA: Burroughs wheeler algorithm
CNV: Copy number variants
DAVID: Database for annotation, visualization and integrated discovery
EAZ: East African Zebu
GATK: Genome analysis toolkit
HLA: Human leukocyte antigen
IUPAC: International union of pure and applied chemists
SIH: Single individual haplotyper
SNP: single nucleotide polymorphism
VCF: Variant call format
